# Intergenomic Rearrangements after Polyploidization of *Kengyilia thoroldiana* (Poaceae: Triticeae) Affected by Environmental Factors

**DOI:** 10.1371/journal.pone.0031033

**Published:** 2012-02-17

**Authors:** Qiuxia Wang, Huitao Liu, Ainong Gao, Xinming Yang, Weihua Liu, Xiuquan Li, Lihui Li

**Affiliations:** 1 The National Key Facilities for Crop Gene Resources and Genetic Improvement (NFCRI), Institute of Crop Sciences, Chinese Academy of Agricultural Sciences, Beijing, People's Republic of China; 2 State Key Laboratory of Special Economic Animal Molecular Biology, Institute of Special Wild Animal and Plant Sciences, Chinese Academy of Agricultural Sciences, Jilin, Jilin, People's Republic of China; Biodiversity Insitute of Ontario - University of Guelph, Canada

## Abstract

Polyploidization is a major evolutionary process. Approximately 70–75% species of Triticeae (Poaceae) are polyploids, involving 23 genomes. To investigate intergenomic rearrangements after polyploidization of Triticeae species and to determine the effects of environmental factors on them, nine populations of a typical polyploid Triticeae species, *Kengyilia thoroldiana* (Keng) J.L.Yang et al. (2n = 6x = 42, StStPPYY), collected from different environments, were studied using genome *in situ* hybridization (GISH). We found that intergenomic rearrangements occurred between the relatively large P genome and the small genomes, St (8.15%) and Y (22.22%), in polyploid species via various types of translocations compared to their diploid progenitors. However, no translocation was found between the relatively small St and Y chromosomes. Environmental factors may affect rearrangements among the three genomes. Chromosome translocations were significantly more frequent in populations from cold alpine and grassland environments than in populations from valley and lake-basin habitats (*P*<0.05). The relationship between types of chromosome translocations and altitude was significant (*r* = 0.809, *P*<0.01). Intergenomic rearrangements associated with environmental factors and genetic differentiation of a single basic genome should be considered as equally important genetic processes during species' ecotype evolution.

## Introduction

Polyploidization is a major evolutionary process that can generate species reproductively isolated from their diploid progenitors. An entirely different trait can result in increased rates of polyploidization and increased evolutionary “success” [1]. Approximately 50–70% of angiosperm species have polyploid origins [2], [3], [4]. Grant [5] proposed that 47% of all flowering plants were of polyploid origin, and that 58% of monocots and 43% of dicots were polyploid. Several reviews concerning polyploidy in plants have covered various aspects including the effects of polyploidy on speciation, genome structure and gene expression [6], [7], [8], [9], [10]. Polyploid species have a greater potential to adapt to a wider range of habitats and demonstrate better survival in unstable climates than their diploid progenitors, suggesting that polyploidy confers enhanced fitness [11], [12], [13]. For example, polyploids are more common than diploids at higher latitudes and altitudes [14].

Chromosome painting (GISH, FISH), genetic mapping, comparative genetics and sequence analysis have provided evidence for intra- and inter-genomic rearrangements in naturally-occurring and synthetic polyploids [6], [9], [15], [16], [17], [18]. Newly-formed polyploid hybrids are not true species unless they are genetically, ecologically, or reproductively isolated from their parental taxa [19]. It is assumed that chromosome rearrangements facilitated the establishment of newly-formed polyploids as successful species [20], [21], [22]. In addition, genome rearrangements result in evolutionary adaptation to environmental changes; extreme phenotypes, which pre-adapt plants for survival in extreme habitats, have resulted from chromosome rearrangements [21], [23]. Nucleocytoplasmic interaction (NCI) could cause chromosome changes after polyploidization, and chromosome changes in turn can restore fertility and nucleocytoplasmic compatibility and facilitate the successful establishment of the new species [6], [24], [25].

For chromosome changes in newly-formed polyploids, NCI is only an internal factor. However, few studies have investigated the correlation between external environmental factors and chromosome changes of polyploids, particularly allopolyploids. Natural allopolyploids provide a model system for studying intergenomic rearrangements of polyploids. Because allopolyploids lack diploid pairing fidelity, recombination may occur ectopically among paralogous or homoeologous sequences, and homoeologous recombination may lead to inter-genomic chromosome rearrangements [26]. Furthermore, the environmental factors present where a natural allopolyploid grows can be measured accurately, and correlations between extensive genome rearrangements and environmental factors can be investigated.

GISH permits entire genomes to be visualized and allows intergenomic rearrangements to be identified. It was utilized to investigate intergenomic rearrangements in *K. thoroldiana*, a natural allopolyploid species of Triticeae. Triticeae is a large taxon with ca. 500 diploid and polyploid species [27]. Within Triticeae, ca. 25–30% species are diploid, and polyploid species account for the remaining 70–75% [28]. All major types of polyploid species, namely autopolyploid, segmental allopolyploid and allopolyploid, are present in this family. The *Kengyilia* genus in Triticeae is one of the best characterized examples of evolution through polyploidization. It is an allohexaploid (2n = 6x = 42, StStPPYY) that arose from hybridization between a tetraploid (2n = 28, StStYY) and a diploid (2n = 14, PP) species, which was subjected to chromosome doubling. The *Kengyilia* genus, predominantly located on the Tibetan Plateau and adjacent areas to the north, includes the St, P and Y genomes, which are very important evolutionary hotspots in Triticeae. The P genome was thought to be independently inherited, and there was no evidence for gene transfer from it to other genomes [29]. The Y genome is rather elusive and its diploid donor is unknown [29], [30].

Genomic changes including intergenomic translocations in resynthesized and natural allopolyploids are common consequences of polyploidization across a wide range of species [9], [26], [31], [32], [33], [34]. Our study demonstrates that genomes do not behave as independent units after polyploidization in nature, but undergo frequent extensive intergenomic rearrangements. In this study, an important factor affecting intergenomic rearrangements was the environment. The frequency and type of genome rearrangements were affected predominantly by ecological factors and altitude.

## Materials and Methods

### Plant materials

Nine populations of *K. thoroldiana* were sampled from Qinghai and Tibet provinces by the Chinese Academy of Agricultural Sciences in 2003. The seeds were collected from individual plants. Fifteen individual plants from one population of *K. thoroldiana* were randomly selected for this study (Table 1). Chromosomes were prepared as described by Wu et al. [35].

**Table 1:** Nine populations of *K. thoroldiana*.

Populations	Altitude(m)	Ecological environment	Group
Z2538	4015	cold alpine	1
Z2627	4062	alpine valley	2
Z2540	4157	alpine salt lake	2
Z2550	4251	lake basin, swamp	2
Z2544	4254	cold alpine grassland	1
Z2541	4273	lake-basin, sediment gravel	2
Z2628	4412	cold alpine hungriness grasslands	1
Z2631	4453	cold alpine hungriness grasslands, salt marsh	1
Z2633	4710	cold alpine grasslands	1

### Labeling of probes

Total genomic DNAs were extracted from young leaves of *Pseudoroegneria spicata* (2n = 2x = 14, StSt), *Agropyron cristatum* (2n = 4x = 28, PPPP), *Triticum aestivum* L. cv. Chinese Spring (2n = 6x = 42, AABBDD) and *Brachypodium sylvaticum* (L.) Beauv., using phenol-chloroform extraction as described by Sharp et al. [36] and modified by Devos et al. [37]. Total genomic DNAs of *Pseudoroegneria spicata* and *Agropyron cristatum* were labeled separately with Biotin-Nick-Translation Mix (Roche, Germany) and Dig-Nick-Translation Mix (Roche, Germany).

### GISH analysis

The two-color GISH method adopted here was described by Nederlof et al. [38] and modified by Han et al. [39], [40] and Sepsi et al. [41]. Genomic DNAs from the Chinese Spring and *B. sylvaticum* were added for blocking purposes, and the probe∶blockers (St: Chinese Spring: *B. sylvaticum*) ratio was 1∶60∶60 according to Wang et al. [42]. The chromosomes of 5–10 cells for each individual were used to identify genotype after GISH.

### Statistical analysis

Tr_fre_ is the frequency of individuals including chromosome translocation in one population (15 individuals in total) or in nine populations (135 individuals in total). CV_j_ is the coefficient of variation of each genotype in one population (15 individuals in total). The synthetic coefficient of variation (CV) of all genotypes was calculated in every population using the following formula modified from Dong [43] and Dong et al. [44]:
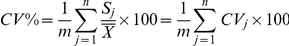
where m equals the number of individuals in a population, n equals the number of genotypes in a population, *S_j_* equals the standard error of each genotype in a population and 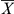 is the mean of each genotype in a population.

The types, numbers and frequencies of St, P and Y chromosome translocations, and CV, were statistically analyzed using Microsoft Excel 2003. Other analyses including two-sample test and correlations were performed using SAS8.0 (SAS Institute, Cary, NC, USA). The ecological environments from which the nine populations were collected were divided into two groups: cold-alpine and grassland (group 1), and valley and lake-basin habitats (group 2) (Table 1). The difference between the means was tested using a two-sample t-test in the two ecological environments. The scatter diagram was drawn using Microsoft® Office Excel 2003. The *P* value of the two-sample test was one-sided, and that of the correlation was two-sided.

## Results

### Analysis of rearrangements among St, P and Y genomes

**Figure 1:**
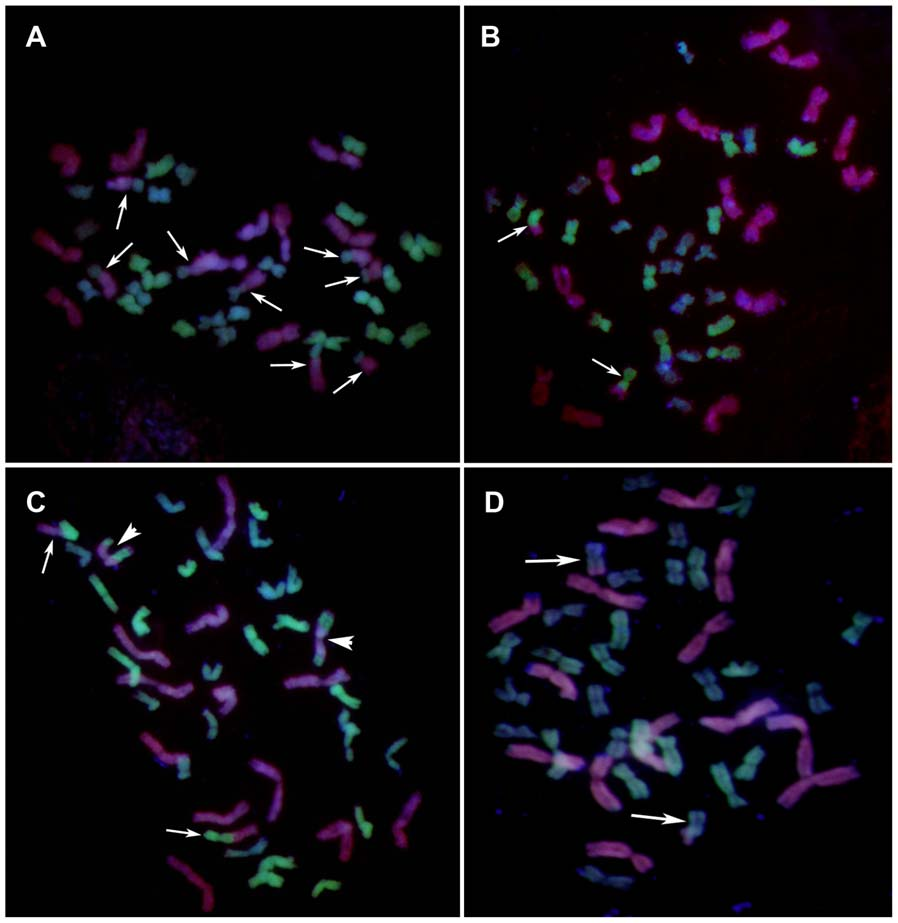
Chromosome translocations of St, P and Y genomes in *K. thoroldiana*. St genome DNA signal is green, P genome DNA signal is red, and blue indicates no hybridization signal. (A) One individual in population Z2538: four pairs of P/Y whole-arm reciprocal translocations (arrow). (B) One individual in population Z2544: two P/St terminal translocations (arrow), 15 P chromosomes and 12 St chromosomes. (C) One individual in population Z2628: one pair of P/St terminal reciprocal translocations (arrow) and one pair of P/St terminal+intercalary reciprocal translocations (arrowhead). (D) One individual in population Z2628: two P/Y terminal translocations (arrow), 14 P chromosomes and 12 Y chromosomes.

A wide range of St, P and Y chromosome translocations were identified using GISH (Fig. 1). The frequency of P/Y chromosomal translocation (22.22%) was significantly higher than that of P/St (8.15%) translocation (two-sample test, Z = 3.22, *P* = 0.0006; Table 2), and no translocation was found between the St and Y chromosomes. Various types were observed; 19.26% were terminal translocations, whole-arm translocations accounted for 11.11%, and intercalary translocations accounted for 1.48% (Table 2). Therefore, rearrangements between the P and Y genomes occurred more often than rearrangements between the P and St genomes. Terminal regions of chromosomes were more active and more involved in rearrangements than centromeric regions.

**Table 2:** Types and frequencies of chromosome translocation in nine populations of *K. thoroldiana*.

Populations	(P/Y)_t_	(P/Y)_w_	(P/St)_t_	(P/St)_i_	Tr_t_	Tr_w_
Z2538	0	15	0	0	0	15
Z2627	0	0	0	0	0	0
Z2540	1	0	0	0	1	0
Z2550	1	0	0	0	1	0
Z2544	6	0	4	0	10	0
Z2541	0	0	1	0	1	0
Z2628	5	0	2	2	7	0
Z2631	0	0	1	0	1	0
Z2633	2	0	3	0	5	0
total	15	15	11	2	26	15
Tr_fre_ (%)	11.11	11.11	8.15	1.48	19.26	11.11
Total Tr_fre_ (%)	22.22		8.15			

(P/Y)_t_: number of individual plants with terminal translocations between chromosomes P and Y;

(P/Y)_w_: number of individual plants with whole-arm translocations between chromosomes P and Y;

(P/St)_t_: number of individual plants with terminal translocations between chromosomes P and St;

(P/St)_i_: number of individual plants with intercalary translocations between chromosomes P and St.

Tr_t_: number of individual plants with all terminal translocations.

Tr_w_: number of individual plants with all whole-arm translocations.

### Types and frequencies of intra- and inter-population genome rearrangements

The frequency of chromosome translocation (Tr_fre_) and synthetic coefficient of variation (CV) were used to characterize the frequency of chromosome translocations and genotype diversity, respectively. Both measures varied greatly inter- and intra-population (Figs. 1A, B; Figs. 1C, D; Table 3). Of the nine populations, the Tr_fre_ of population Z2538 was the highest (100%), while the genotype diversity was highest in population Z2633, with CV = 70.33% (Table 3). No chromosomal translocation was found in population Z2627 (Table 3).

**Table 3:** Genotype diversity and frequencies of translocations in nine populations of *K. thoroldiana*.

populations	genotype	total	CV_j_	CV(%)	Tr	Tr_fre_
Z2538	8(P/Y)_w_	15	0.00	0.00	15	100
Z2627	Ntr	15	0.00	0.00	0	0
Z2540	8(P/Y)_t_	1	3.87			
	Ntr	14	0.28	27.67	1	6.67
Z2550	4(P/Y)_t_	1	3.87			
	Ntr	14	0.28	27.67	1	6.67
Z2544	4(P/Y)_t_	6	1.27			
	2(P/St)_t_+14P	3	2.07			
	2(P/St)_t_+15P	1	3.87			
	Ntr	5	1.46	57.80	10	66.67
Z2541	4(P/St)_t_	1	3.87			
	Ntr	14	0.28	27.67	1	6.67
Z2628	2(P/Y)_t_	4	1.72			
	4(P/Y)_t_	1	3.87			
	2(P/St)_t_+2(P/St)_i_	2	2.64			
	Ntr	8	0.97	61.33	7	46.67
Z2631	2(P/St)_t_	1	3.87			
	Ntr	14	0.28	27.67	1	6.67
Z2633	2(P/Y)_t_	1	3.87			
	4(P/Y)_t_	1	3.87			
	4(P/St)_t_	3	2.07			
	Ntr	10	0.73	70.33	5	33.33

8(P/Y)_w_: 8 whole-arm translocations between chromosomes P and Y.

Ntr: no chromosome translocations.

2(P/St)_t_+2(P/St)_i_: 2 terminal translocation and 2 intercalary translocations between chromosomes P and St.

Tr: number of individual plants with all types of translocations.

The mean Tr_fre_ of populations collected from group 1 was 50.67%, significantly higher than the 5.00% from group 2 populations (two-sample t-test, t value = 2.89, df = 4, *P* = 0.04). A two-sample t-test demonstrated no significant difference between the mean CVs for groups 1 and 2 (*P*>0.05). Generally, the Tr_fre_ of populations located in cold alpine or grassland habitat was higher than that of populations located in a valley or lake-basin grassland (e.g. Z2538 and Z2627; Table 3). However, there was no correlation between CV and ecological environment.

**Figure 2:**
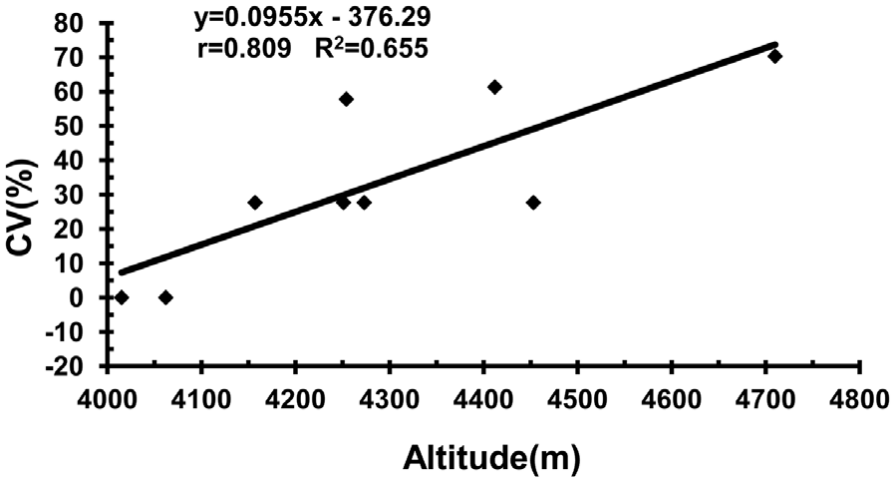
Scatter plot of altitude against CV. Each dot represents a module.

Correlations between Tr_fre_ and altitude, latitude and longitude were not significant. However, the relationship between CV and altitude was highly significant (*r* = 0.809, *P* = 0.008; Fig. 2). Genotype diversity is higher in general with increasing altitude. At low altitude, chromosome translocations were observed between the P and Y genomes, as demonstrated in populations Z2538, Z2540 and Z2550 (Table 2 and 3). P/St translocations appeared with increasing altitude, and the types of translocation became more complex and the genotype diversity increased, as demonstrated in populations Z2544, Z2628 and Z2633 (Table 2 and 3). However, there were no significant correlations between CV and latitude or longitude.

## Discussion

### Rearrangements among St, P and Y genomes after polyploidization

Genomes were not independent after polyploidization. There were P/Y and P/St intergenomic chromosome translocations, but no St/Y chromosome translocation was found in *K. thoroldiana*. P/Y chromosomal translocations were significantly more frequent than P/St in the present study. The P genome is larger than the St and Y genomes [42]. Yen et al. [45] suggested that the *Kengyilia* genus originated from an allohexaploid hybridization formed between a diploid (2n = 14, PP) and a tetraploid (2n = 28, StStYY) species. The present study demonstrated that rearrangements occurred between the large P genome and small St and Y genomes when the allohexaploid hybridization formed. Also, the St and Y genomes were inherited from the same parent, explaining why there was no translocation between them. The results suggest that intergenomic rearrangements occurred after polyploidization, confirming the origin of the *Kengyilia* genus previously reported by Yen et al. [45]. Hybrid breeding between common wheat and wild relative species in Triticeae is an example of polyploidization. And intergenomic rearrangements after polyploidization provide a basis for the transfer of desirable genes from perennial Triticeae grasses into wheat.

New reorganized genomes occur after polyploidization. Dewey [29] suggested that the P genome was independently inherited, and that there was no gene transfer from the P genome to others. Accordingly, Jensen and Griffin [46] thought it would be impossible to transfer genes from *Agropyron* (P genome) to cereals without biotechnology. However, this study indicates that the P genome did not behave independently but was very active and subject to natural rearrangements with other genomes after polyploidization. Such intergenomic rearrangements could lead to the production of reorganized genomes that would not occur in the diploid state, making it more difficult to identify the diploid donors of reorganized genomes in allopolyploid species. The B genome of wheat is thought to be an example of such a reorganized genome [47]. The supposed diploid donor of the Y genome in Triticeae is unknown [29], [30], and the genome is always present in polyploid species. The Y genome could be a reorganized genome similar to the B genome of wheat, evolving via rearrangement after polyploidization. There was little fragmental intergenic exchange and introgression within the St, P and Y chromosomes of naturally occurring polyploid [42]. Therefore, the genomes of polyploids are different from those of their diploid donors. Distinct methods should be applied to investigate polyploid genomes and their diploid donor genomes, which could ultimately lead to improved definition of the origin and evolution of species.

The results of this study demonstrate that the terminal regions of chromosomes are more actively involved in rearrangements than centromeric regions. Accordingly, P/St intercalary translocations occurred on terminal regions (Fig. 1). Some chromosomes, regions, or sites are more likely to be subject to translocation than others [48]. The terminal regions of the St, P and Y chromosomes are more susceptible to translocation than centromeric regions, as demonstrated by Wang et al. [42]. The frequency of intercalary translocation was the lowest; only one individual in the nine populations of *K. thoroldiana* utilized in this study included an intercalary translocation (Fig. 1C, Table 2). Mu et al. [49] inferred that intercalary translocation was related to chromosome breakage and reunion. It requires more chromosome breakage events than terminal and whole-arm translocations, and therefore rarely occurs.

### Types and frequencies of intergenomic rearrangements in nine populations from different environmental factors

Nine natural populations of *K. thoroldiana* collected from various environments were used to study the correlations between the environmental factors and genome rearrangements in our study. The frequencies and types of chromosomal translocations at the intra- and inter-population levels were varied. Genome rearrangements were more frequent in populations collected from a cold alpine and grassland environment than in populations from a lake-basin or valley (Table 3). Weidema et al [50] inferred that a feature maintaining high genetic diversity in the populations could be the high outcrossing rate. In the grassland environment, ambulation of humans and grazing livestock coupled with wind caused more effective pollen spreading and genetic flow than in valley and lake-basin habitats. Weidema et al. [50] also conclude that wind pollination is possible and together with insect pollination is causing the observed patterns of genetic variation; and the species occurs on open and very exposed sites, where wind pollination is effective. Cold alpine and grassland are relatively more open and wind-exposed than lake-basin or valley. Pollinators could be useful for increasing the frequency of cross-pollination, which could in turn lead to hybridization and polyploidization. Owing to adverse interactions between nuclear and cytoplasmic genomes, chromosome changes took place soon after hybridization and facilitated the successful establishment of the newly-formed hybrid [24], [25]. Brysting et al. [25] suggested that intergenomic chromosome changes may play an important role in the evolution of natural hybrids and the establishment of new evolutionary lineages. Together, genome rearrangements of *K. thoroldiana* in a cold alpine and grassland environment may be more than that in a lake-basin or valley.

*K. thoroldiana* is the most advanced group in the phylogeny of the *Kengyilia* genus, and is distributed in the highest altitude of the Tibetan Plateau region [51]. After polyploidization, certain chromosomal changes must take place in the nuclear genome to restore fertility and nucleocytoplasmic compatibility [6], [24], [25]. Also, the complex and changeable environment of the Tibetan Plateau region may affect polyploid genomes in plateau plants. Grun [52] inferred that chromosome pairing was disturbed when plants were placed under abnormal conditions. He also found more univalent frequency in pollen mother cells of most divisions of the plants grown in an alpine garden at 3050 m than in divisions of the same plants grown in gardens at 1400 m and 30 m. The Tibeteau Plateau, with an average altitude of nearly 5000 m and low temperatures (mean annual temperature over most of the region falling below 0°C), has been called the “third pole” of the earth [53], while solar radiation on the plateau is extremely high. The intensities of heat and cold sources, temperature, wind, stability, and the weather patterns reveal characteristic seasonal variations and some remarkable daily variations [54]. According to conclusion of Grun [52], the complex and changeable environment of the Tibetan Plateau region may disturb normal chromosome pairing in meiosis in *K. thoroldiana*, consequently leading to genome rearrangements. This implies a correlation between genome rearrangements and environmental factors. The environment becomes more complex and changeable with altitude, and the interference may become correspondingly stronger. Genome rearrangement was more complex in the populations collected at high altitude than that in populations collected at low altitude in our study. Alternatively, there may be complex genome rearrangements in all nine populations after polyploidization, but individuals showing some types of genome rearrangements could not survive in some environments, while others could survive in a more complex environment. Whatever it was, natural selection plays a role in the establishment and maintenance of genome rearrangement after polyploidization. It has also been suggested that natural selection is the major determinant of both RAPD and allozyme diversities, both being correlated with environmental stress [55].

In the present study, it was evident that diverse intergenomic rearrangements occurred due to chromosomal translocations, and there was a correlation between genome rearrangements and environmental factors. Genomes did not behave as independent units after polyploidization. Intergenomic rearrangements associated with environmental factors and genetic differentiation of a single basic genome should be considered as equally important genetic processes during ecotype evolution. The genomes of plateau plants are not only affected by nucleocytoplasmic compatibility, but also by environmental factors. The complex environment could affect genome rearrangement, which facilitates the successful establishment of the newly-formed hybrid. Natural selection plays a role in the establishment and maintenance of new species after polyploidization.
